# Assessment of Healthcare Professionals’ Knowledge and Practices About Radioactive Iodine Preparation in Thyroid Cancer Patients

**DOI:** 10.7759/cureus.72837

**Published:** 2024-11-01

**Authors:** Raghad S Alzahrani, Suzana Ezzi, Mohammed M Alghamdi, Ali S Alsudais, Nawal Yahya, Fetoon M Aljuaid, Suhaib Radi

**Affiliations:** 1 College of Medicine, King Saud Bin Abdulaziz University for Health Sciences, Jeddah, SAU; 2 College of Medicine, King Abdullah International Medical Research Center, Jeddah, SAU; 3 Endocrinology, King Abdulaziz Medical City, Jeddah, SAU

**Keywords:** healthcare providers' knowledge, healthcare providers’ practices, radioactive iodine, radioactive iodine preparation, thyroid cancer

## Abstract

Background

Radioactive iodine (iodine-131) therapy is widely used for treating thyroid cancer. However, the knowledge and practices of healthcare professionals in preparing patients for radioactive iodine therapy may vary, potentially affecting patient outcomes.

Objective

This study aimed to evaluate the knowledge and practices of healthcare professionals at King Abdulaziz Medical City (KAMC) in Jeddah, regarding the preparation of patients with thyroid cancer for radioactive iodine therapy.

Methodology

A cross-sectional study was conducted using an electronic survey distributed to endocrinology consultants, fellows, medical residents, radiologists, nuclear medicine technicians, and nurses at KAMC. The survey assessed participants' knowledge and practices related to radioactive iodine preparation. Using JMP 17 database software (JMP, Cary, NC), descriptive and inferential statistical analyses were performed and qualitative data were analyzed thematically.

Results

The study included 30 participants, half of whom had five years of experience or less (43.33%, n=13). More than half of the respondents reported involvement in the administration of iodine-131 (56.67%, n=17). Only around half the participants correctly identified certain aspects, such as the need for recombinant human thyroid stimulating hormone (TSH) prior to iodine therapy (56.67%, n=17), adherence to a low-iodine diet (43.33%, n=13), screening female patients for pregnancy (76.67%, n=23), the duration for which women should avoid pregnancy (56.67%, n=17) and patient education on the side effects and distance precautions when sleeping with others (48%, n=12). The study found a significant association between involvement in the administration or supervision of iodine-131 and knowledge of the necessary medication prior to treatment (p=0.0431 for correct choice and p=0.0117 for correct dosage).

Conclusion

The study revealed knowledge gaps among healthcare professionals at KAMC regarding the preparation of patients with thyroid cancer for radioactive iodine therapy. It emphasizes the need for educational and training initiatives to improve healthcare professionals' knowledge and awareness in this area. Addressing these knowledge gaps can enhance patient safety and optimize the effectiveness of radioactive iodine therapy.

## Introduction

Thyroid cancer and hyperfunctioning thyroid disease can both be treated with radioactive iodine (iodine-131) therapy (RAI), which is categorized as radioactive nuclear medicine [1,2]. It was first synthesized in 1941 and received Food and Drug Administration (FDA) approval in 1971 for its therapeutic use [3,4]. Iodine, which is a natural precursor for the thyroid hormones triiodothyronine (T3) and thyroxine (T4), is taken up from the blood and delivered to the thyroid follicular cell by the sodium and iodide transporters. Similarly, when RAI is absorbed by the thyroid's follicular cells, it emits beta rays that further inflict permanent local damage on the tissue [5-8]. Gamma rays that radioactive iodine emits are beneficial for diagnostic purposes. The use of this radiation as a diagnostic tool can help distinguish between thyroid diseases that are either hyperactive or hypoactive. This separation happens by estimating the take-up of radioactive iodine in the thyroid after 24 hours [9-11].

Research has demonstrated that RAI significantly enhances survival rates in patients of Arab ethnicity with differentiated thyroid cancer (DTC), as well as for individuals of other ethnicities, across both adult and pediatric populations [7]. RAI for hyperthyroid patients is recommended for people who are at high risk for surgery and have a shorter life expectancy, who haven't reached an euthyroid status, or who can't take oral anti-thyroid medications [8]. On the other hand, it is contraindicated in pregnancy, breastfeeding, moderate to severe Graves' ophthalmology, and severe thyrotoxicosis [10,12]. RAI is ineffective in patients who have a hyperfunctioning disease without iodine uptake because its action is dependent on iodine uptake from thyroid tissue. In patients with thyroid carcinoma, RAI can be given four to six weeks after surgery as an adjuvant therapy [8]. Treatment with RAI may predispose patients to permanent hypothyroidism because it causes definitive destruction of thyroid tissue, which might require lifelong replacement therapy [9,10]. Furthermore, bone marrow depression, leukemia, stomach cancer, bladder cancer, and breast cancer could be late complications of RAI [2].

Patients must have a high level of thyroid-stimulating hormone (TSH or thyrotropin) in the blood in order for the thyroid tissue (and cancer cells) to take up radioactive iodine. That’s why it’s important to prepare patients properly before starting RAI. The practice of differentiated thyroid cancer (DTC) has been transformed by the development of recombinant human TSH. As part of the dynamic risk assessment process, it plays a crucial role in evaluating the risk of recurrence post-treatment in addition to playing a well-established role in RAI ablation preparation [13,14]. This is done by facilitating a stimulated thyroglobulin level (in the absence of a highly sensitive thyroglobulin assay). Furthermore, it’s recommended to follow a low-iodine diet for one to two weeks prior to therapy [15,16]. This entails avoiding foods like dairy, eggs, seafood, soy, and red dye as well as iodized salt.

There are some precautions that should be taken into consideration for RAI therapy. For example, women who are pregnant or nursing should not receive RAI therapy [17, 18]. Additionally, after RAI, patients should avoid prolonged close physical contact with others, especially children and pregnant women, and they should sleep alone in a separate bed [19]. Personal items should be brought home by patients and washed separately from other belongings before usage. Moreover, patients should be urged to take daily showers and to consume a lot of water while they are under restrictions [20]. Therefore, RAI use should be under the supervision of an interprofessional team, including an endocrinologist, a primary care clinician, nuclear medicine staff, nurses, and pharmacists. Nurses and other healthcare professionals who work directly with patients should be knowledgeable about the preparations and precautions of RAI. This cross-sectional research aims to assess healthcare professionals' knowledge and practice about radioactive iodine preparation in patients with thyroid cancer and hyperthyroidism.

## Materials and methods

Study objective 

This study aimed to assess the practices and knowledge of healthcare practitioners at King Abdulaziz Medical City (KAMC) in Jeddah regarding the preparation of radioactive iodine in patients with thyroid cancer and hyperthyroidism. The specific objectives of the study were to evaluate the healthcare practitioners' knowledge regarding radioactive iodine preparation, estimate the prevalence of inadequate knowledge among healthcare practitioners, and explore the most prevalent misconceptions related to radioactive iodine preparation.

Setting and participants 

The study was conducted at KAMC, a renowned healthcare institution established in July 1982, employing a substantial workforce of over 8000 individuals. The study participants comprised endocrinology consultants and fellows, medical residents, radiologists, nuclear medicine technicians, and nurses working in clinics and wards where patients received radioactive iodine treatment at KAMC in Jeddah. No specific exclusion criteria were applied for participant selection, ensuring inclusivity across various healthcare roles.

Study design

A cross-sectional study design was employed to collect data for this research endeavor. An electronic survey was utilized as the primary data collection instrument, distributed via email to reach all eligible participants. Convenience sampling was employed to select participants, allowing for the inclusion of readily available and accessible healthcare practitioners, thereby enhancing the feasibility of the study.

Data collection 

Data collection was carried out by the authors using an online self-administered questionnaire. The research team developed the questionnaire using Google Form, ensuring its relevance and validity to the study objectives. Participants were invited to complete the questionnaire electronically, with email serving as the distribution channel. The collected data were then organized using Excel software. The questionnaire comprehensively assessed the knowledge and practices of healthcare practitioners regarding the preparation of radioactive iodine in thyroid cancer patients.

Data analysis

Upon completion of the data collection phase, the collected questionnaires were analyzed using JMP 17 database software (JMP, Cary, NC). Descriptive and inferential statistical analyses were employed to examine the quantitative data, with a p-value threshold of 0.05 used to reject the null hypothesis. The types of tests used for associations included chi-square and Fisher's exact tests. Continuous variables were summarized using means and standard deviations, while categorical variables were presented as percentages. Furthermore, the qualitative data collected through the survey underwent thematic analysis to provide deeper insights and explanations that complemented the quantitative findings.

Ethical considerations

Ethical considerations were diligently addressed throughout the study. Prior to data collection, the research team sought approval from the Institutional Review Board (IRB), which was granted (NRJ23J/062/02) to ensure compliance with ethical guidelines. Confidentiality and participant anonymity were ensured by refraining from collecting personal identifying information such as names or phone numbers. Informed consent was obtained from all the participants before their engagement in the study, and participants had the autonomy to withdraw from the research at any point without repercussions. Voluntary participation was emphasized throughout the study. All data collected were securely stored within the premises of KAMC and made accessible exclusively for research purposes.

## Results

Demographical data

A total of 30 respondents were included in the study, consisting of 17 females (56.67%) and 13 males (43.33%). Around half the participants fell within the age group of 31-40 years (46.67%, n=14), followed by those aged 30 or younger (40.00%, n=12). The largest percentage of participants (43.33%, n=13) reported having five years or less of professional experience, while only three participants (10.00%) had 16 years or more of experience. The participants were from various professional backgrounds, with the most represented group being endocrinologists (43.33%, n=13), followed by internists (20.00%, n=6) and nurse practitioners (13.33%, n=4). Some participants held an MS/PhD or BA degree (53.55%, n=16; 40%, n=12). In terms of their affiliated healthcare facilities, the majority worked in internal medicine (46.67%, n=14) or outpatient departments (43.33%, n=13) (Table 1).

**Table 1:** Participants’ Knowledge Regarding Administration of Radioactive Iodine Therapy *Correct answer

Diet to be followed before commencing therapy	n (%)
Low iodine*	13 (43.33%)
Low salt	2 (6.67%)
No special diet needed	2 (6.67%)
Low protein	1 (3.33%)
“I don’t know”	12 (40.00%)
Medications to be administered before commencing therapy	n (%)
Recombinant human TSH*	17 (56.67%)
Beta-blockers	1 (3.33%)
None is needed	1 (3.33%)
“I don’t know”	11 (36.67%)
Number of doses needed of the chosen medication prior to therapy	n (%)
One dose	1 (3.33%)
Two doses*	13 (43.33%)
No medication needed	5 (16.67%)
“I don’t know”	11 (36.67%)
Route of administration of therapy	n (%)
Intramuscular	4 (13.33%)
Intravenous	1 (3.33%)
Oral*	17 (56.67%)
Subcutaneous	1 (3.33%)
“I don’t know”	7 (23.33%)
Screening for pregnancy prior to commencing therapy	n (%)
Always*	23 (76.67%)
Frequently/Not always	1 (3.33%)
“I don’t know”	6 (20.00%)
Duration of time recommended for female patients to avoid pregnancy after finishing therapy	n (%)
3 months	1 (3.33%)
6 months*	9 (30.00%)
12 months*	8 (26.67%)
“I don’t know”	12 (40.000%)
Duration of time recommended for male patients to avoid fathering a child after finishing therapy	n (%)
1 month	1 (3.33%)
3 months*	5 (16.67%)
6 months*	5 (16.67%)
12 months	3 (10.00%)
None needed	4 (13.33%)
“I don’t know”	12 (40.00%)
Is consent needed prior to therapy?	n (%)
Yes*	19 (63.33%)
No	1 (3.33%)
“I am not involved in the administration of therapy”	10 (33.33%)

Participant knowledge and perceptions

The survey distributed to participants involved a series of questions to measure the participants’ general knowledge about the safety and efficacy of administering RAI for thyroid disorders. More than half of the participants (56.67%, n=17) reported being involved in the administration or supervision of RAI for thyroid cancer/hyperthyroidism. When asked about whether a medication is needed to be administered prior to iodine therapy, 56.67% (n=17) have correctly identified rhTSH as the required agent, while 43.33% (n=13) have also chosen the correct number of doses required for administration beforehand. Similarly, more than half of the participants have also correctly identified the oral route as the method of administration (56.67%, n=17) and low iodine as the preferred diet for patients undergoing RAI therapy (43.33%, n=13) (Table 2).

**Table 2:** Knowledge among participants about precautionary measures based on radioactive iodine dose

Type of precaution	Radioactive iodine dose, mCi (MBq)					
	Number of responses, % (n)					
	15 (555)	30 (1110)	100 (3700)	150 (5550)	300 (111,000)	“I don’t know”
Patient should sleep in a separate bed from adults	N/A	26.67% (8)	23.33% (7)	N/A	N/A	50.00% (15)
Patient should sleep in a separate bed from children and pregnant women	13.33% (4)	23.33% (7)	10.00% (3)	N/A	N/A	53.33% (16)
Patient could return immediately to work	17.24% (5)	24.14% (7)	N/A	N/A	N/A	58.62% (17)
Patient should maximize the distance (6 feet) from children and pregnant women	3.33% (1)	26.67% (8)	6.67% (2)	3.33% (1)	N/A	60.00% (18)
Patient should avoid extended time in public areas	N/A	16.67% (5)	20.00% (6)	3.33% (1)	N/A	60.00% (18)
Patient should avoid public transportation	13.33% (4)	10.00% (3)	23.33% (7)	N/A	N/A	53.33% (16)
Patient should avoid sexual contact	16.67% (5)	26.67% (8)	3.33% (1)	N/A	N/A	53.33% (16)
Patient should avoid contacting children less than 2 years	16.67% (5)	20.00% (6)	10.00% (3)	N/A	N/A	53.33% (16)
Patient should avoid contacting children between 2-10 years	13.33% (4)	20.00% (6)	13.33% (4)	N/A	N/A	53.33% (16)
Patient should dispose of toothbrush	16.67% (5)	20.00% (6)	10.00% (3)	N/A	N/A	53.33% (16)
Patient should wash bedding and clothes separate from family	20.00% (6)	16.67% (5)	6.67% (2)	3.33% (1)	N/A	53.33% (16)
Patient should wash dishes separate from family	13.33% (4)	23.33% (7)	6.67% (2)	N/A	N/A	56.67% (17)
Patient does not need to be hospitalized	23.33% (7)	16.67% (5)	6.67% (2)	N/A	N/A	53.33% (16)
Patient should be hospitalized for 24 hours	10.00% (3)	10.00% (3)	23.33% (7)	3.33% (1)	N/A	53.33% (16)
Patient should be hospitalized for 48 hours	10.00% (3)	6.67% (2)	16.67% (5)	13.33% (4)	3.33% (1)	50.00% (15)
Patient should be hospitalized for 72 hours	10.00% (3)	10.00% (3)	10.00% (3)	10.00% (3)	6.67% (2)	53.33% (16)
Patient should stay in a hotel to avoid contamination at home	13.33% (4)	6.67% (2)	20.00% (6)	N/A	3.33% (1)	56.67% (17)
Patient should not use this dose	20.00% (6)	6.67% (2)	6.67% (2)	N/A	6.67% (2)	60.00% (18)

Furthermore, on the screening-related sections, 76.67% (n=23) stated that they screen female patients for pregnancy prior to administration of therapy, with 66.67% (n=20) using urine or serum pregnancy test results as a standard. Moreover, 17 participants (56.67%) reported the correct number of months for which women should avoid pregnancy, where answering six or 12 months was considered correct. On the other hand, only 33.33% (n=10) answered correctly as the recommended duration for which male patients should avoid fathering children, where three or six months were considered correct answers.

Another part of the questionnaire was to assess the extent to which healthcare providers have provided counseling and education to their patients prior to undergoing iodine therapy. The highest number of participants (40.00%, n=12) confirmed always educating their patients about the side effects of the medication and, as a precaution, the distance required when sleeping aside from other members. This was followed by always providing education regarding precautions to have contact with children or women (36.67%, n=11) and then always educating about bathroom use (33.33%, n=10). However, the fewest (26.67%, n=8) have stated always providing education regarding the normal structure and function of the gland.

Regarding the section designed to measure the subjects’ knowledge about the precautions with different doses of therapy, a substantial percentage of participants reported lacking sufficient knowledge to choose an answer. All questions displayed from 50.00% to 60.00% of participants choosing “I don’t know” as their answer choice, as displayed in Table 3.

**Table 3:** The association between different healthcare professionals and the medication choices prior to RAI (radioactive iodine) administration Abbreviations: rhTSH: recombinant human thyroid stimulating hormone; LT4: levothroxine.

Healthcare profession	rhTSH	Beta blockers	I don't know	None is needed	Other: Depend on cause of RAI if for Ca thyrogen or LT4
Endocrinologists (n=13)	11 (84.62%)	0	0	1 (7.69%)	1 (7.69%)
Internists (n=6)	2 (33.33%)	0	4 (66.67 %)	0	0
Nurse practitioners (n=4)	2 (50%)	0	2 (50%)	0	0
Medical intern (n=1)	0	0	1 (100%)	0	0
Nuclear medicine physician (n=1)	1 (100%)	0	0	0	0
Oncologist (n=1)	0	0	1 (100%)	0	0
Pharmacist (n=3)	0	1 (33.3%)	2 (66.667%)	0	0
Registered nurse (n=1)	0	0	1 (100%)	0	0
Total (n=30)	16 (53.33%)	1 (3.33%)	11(36.7%)	1 (3.33%)	1 (3.33%)

Associations between answers and demographics

**Figure 1:**
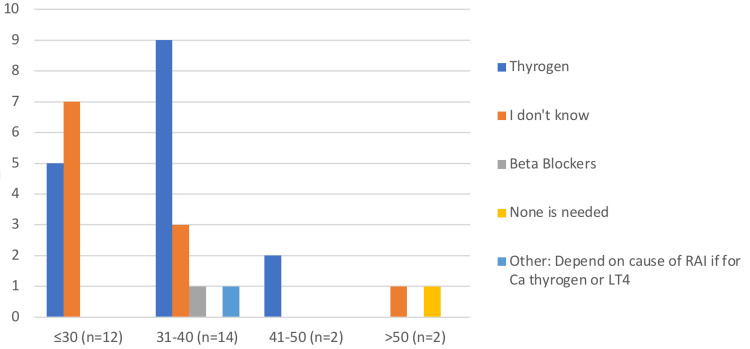
The association between age and the selection of the appropriate medication to be given prior to RAI Image credit: Mohammed M. Alghamdi LT4: levothroxine

A substantial correlation (p=0.037) was discovered in our study investigating the relationship between age and the selection of the appropriate medication (rhTSH) to be given prior to RAI administration (Figure 1). Out of 30 respondents divided into four age categories (≤30, 31-40, 41-50, and ≥51 years), 53.33% selected rhTSH. Among the ≤30 age group, 58.33% selected "I don’t know" and 41.67% correctly selected rhTSH, with none choosing "beta blockers" or "none is needed". In the 31-40 age group, 7.14% chose "beta blockers", 21.43% selected "I don't know" and 64.29% correctly chose rhTSH. All participants in the 41-50 age group correctly chose rhTSH, with none selecting other options. However, those aged ≥51 years split their choices evenly between "I don't know" and "None is needed", with none selecting rhTSH, beta-blockers, or other.

The study analyzed the relationship between different healthcare professionals and medication choices prior to RAI administration (Table 4). The professionals include endocrinologists, internists, medical interns, nuclear medicine physicians, nurse practitioners, oncologists, pharmacists, and registered nurses (RNs). Among endocrinologists, there were a total of 13 responses. The medication choices included 11 (84.62%) correctly selecting Thyrogen, 1 (7.69%) selecting "Other: Depend on cause of RAI if for Ca Thyrogen or LT4", and one (7.69%) selecting "None is needed". For internists, out of six responses, two (33.33%) chose Thyrogen, four (66.67%) chose I don’t know, and none chose beta-blockers, or "None is needed". Medical interns had only one response, and Thyrogen was selected. Nuclear medicine physicians had one response as well, and the choice was "I don’t know". Nurse practitioners had four responses. Among them, two (50%) selected Thyrogen and the other two (50%) chose "I don’t know". The only oncologist picked "I don’t know". Pharmacists had three responses. Out of those, two (66.67%) chose "I don’t know" and one (33.33%) chose beta-blockers. The registered nurse (RN) had one response, which was "I don’t know". In total, the medication choices included 16 (53.33%) rightly selecting Thyrogen, 1 (3.33%) selecting beta-blockers, one (3.33%) selecting Other: "Other: Depend on cause of RAI if for Ca Thyrogen or LT4", 11 (36.7%) selecting "I don't know", and one (3.33%) selecting "None is needed". The statistical analysis, which utilized chi-square and Fisher's exact test, did not reveal a significant association between professional type and medication choice (p=0.4184), implying that various medication choices were made across different healthcare professionals.

**Table 4:** The association between drug dosage selections of rhTSH and different healthcare practitioners

Healthcare Practitioner	2 Doses	1 Dose	I don't know	No response
Endocrinologists (n=13)	12 (92.31%)	0	0	1 (7.69%)
Internists (n=6)	0	0	5 (83.33%)	1 (16.67%)
Medical Intern (n=1)	0	0	1 (100%)	0
Nuclear Medicine Physician (n=1)	1 (100%)	0	0	0
Nurse Practitioners (n=4)	0	1 (25%)	1 (25%)	2 (50%)
Oncologist (n=1)	0	0	1 (100%)	0
Pharmacist (n=3)	0	0	3 (100%)	0
Registered Nurse (RN) (n=1)	0	0	0	1 (100%)
Total (n=30)	13 (43.33%)	1 (3.33%)	11 (36.67%)	5 (16.67%)

**Figure 2:**
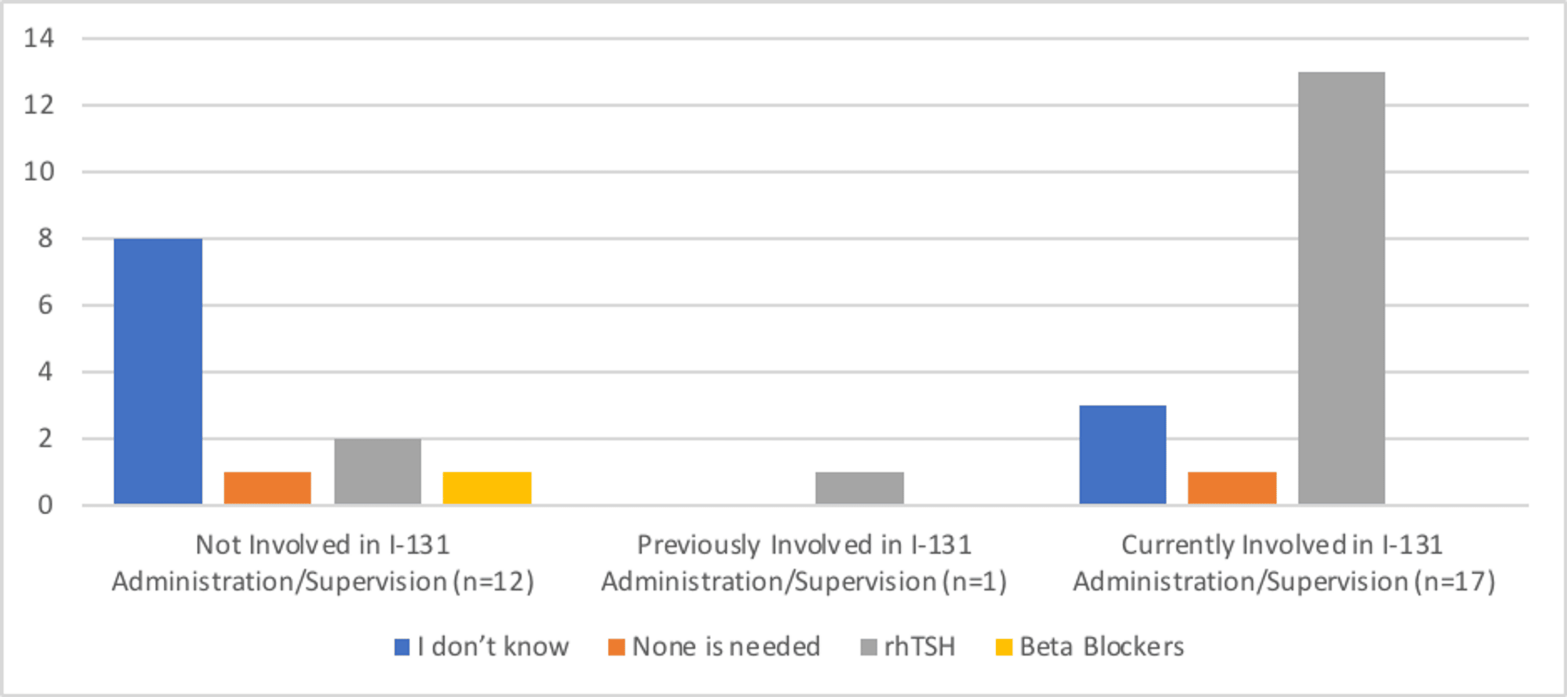
The association between involvement in administration/supervision of I-131 and choice of medication Image credit: Mohammed M. Alghamdi

In addition to the medications needed before RAI treatment, our survey measured participants' involvement in and knowledge of the administration of I-131 for treating thyroid cancer and hyperthyroidism. Out of the 12 respondents who were not involved in administering or supervising I-131, eight were unaware of the required medication, one thought none was required, and two advised correctly using rhTSH. The single respondent who was previously involved advised the use of rhTSH. Moreover, three of the 17 participants who are currently involved did not know about the required medication, one thought none was required, and 13 suggested the correct choice which is using rhTSH. The association between the involvement in administration or supervision of I-131 and knowledge about the necessary medication prior to RAI (Figure 2) was found to be statistically significant (p=0.0431).

The statistical analysis tested the association between drug dosage selections of rhTSH and different healthcare practitioners, where "two doses" is identified as the correct choice. Notably, out of 13 endocrinologists, 92.31% correctly chose "two doses". Contrarily, five of the six internists demonstrated a lack of certainty by choosing "I don't know" and one with no response. The single medical intern and the nuclear medicine physician selected "I don't know" and "two doses", respectively. Among the four nurse practitioners, their responses varied, with one choosing "one dose", one responding with "I don't know" and two abstaining from an answer. The only oncologist and the three pharmacists all responded with "I don't know", while the single RN did not provide an answer. Overall, of the 30 participants, 43.33% rightly identified "two doses", 36.67% expressed uncertainty, and 16.67% refrained from responding. The p-value of 0.0020, which is below 0.05, signifies a statistically significant variance in dosage selection among these professional categories, indicating a need for additional education, especially for non-endocrinologists (Table 5).

**Table 5:** The association between drug dosage selections of rhTSH and different healthcare practitioners

Healthcare Practitioner	Two Doses	One Dose	I don't know	No response
Endocrinologists (n=13)	12 (92.31%)	0	0	1 (7.69%)
Internists (n=6)	0	0	5 (83.33%)	1 (16.67%)
Medical Intern (n=1)	0	0	1 (100%)	0
Nuclear Medicine Physician (n=1)	1 (100%)	0	0	0
Nurse Practitioners (n=4)	0	1 (25%)	1 (25%)	2 (50%)
Oncologist (n=1)	0	0	1 (100%)	0
Pharmacist (n=3)	0	0	3 (100%)	0
Registered Nurse (RN) (n=1)	0	0	0	1 (100%)
Total (n=30)	13 (43.33%)	1 (3.33%)	11 (36.67%)	5 (16.67%)

## Discussion

This cross-sectional study aimed to evaluate the knowledge and practices of healthcare professionals regarding the preparation of RAI in patients diagnosed with thyroid cancer. A sample of 30 participants, consisting of endocrinologists, internists, and nurse practitioners, was included in the study. The results revealed that around half the participants demonstrated a satisfactory understanding of important aspects related to I-131 administration, including the recommended dietary guidelines preceding RAI, as well as the appropriate type, dosage, and route of medication administration prior to RAI. Moreover, participants recognized the significance of screening patients for pregnancy before initiating RAI treatment. However, notable deficiencies in knowledge were observed concerning precautionary measures with specific RAI doses, such as the need for patients to sleep separately from others. Overall, the study underscores the critical role of healthcare professionals' knowledge and adherence to established protocols for RAI preparation to ensure patient welfare and achieve optimal treatment outcomes. Further educational interventions and standardization of guidelines are warranted to address the identified knowledge gaps and enhance the overall quality of patient care in this domain.

A comparable investigation was conducted to assess the understanding of healthcare professionals regarding radiation safety. The results revealed that their knowledge about ionizing radiation and the doses involved in radiological examinations was quite poor. The correct response rates for physicians, nurses, medical technicians, and other personnel groups were approximately 15.7±3.7, 13.0±4.0, 10.1±2.9, and 11.8±4.0, respectively [21]. A separate investigation conducted in Tanzania revealed a lack of adequate understanding among clinicians regarding the appropriate management and utilization of RAI in patients with differentiated thyroid cancer (DTC). The study found that only 7.30% of surgeons were knowledgeable about the significance of thyroxine therapy or RAI ablation following surgery [22].

The study findings have important implications for inter-professional collaboration in the context of RAI therapy. A successful implementation of RAI therapy requires the involvement of various healthcare professionals, including endocrinologists, primary care clinicians, nurses, and pharmacists. It is crucial that these professionals have a solid understanding of RAI preparation protocols to ensure coordinated and effective patient care. The study findings indicate the need for continuous education and training programs that promote interdisciplinary collaboration and enhance the knowledge and skills of healthcare professionals involved in RAI therapy. By fostering collaboration and improving knowledge sharing, the quality of care for patients with thyroid cancer can be significantly enhanced.

One of the strengths of the study is its comprehensive assessment of healthcare professionals' knowledge and practices regarding radioactive iodine preparation in patients with thyroid cancer. The study included a diverse range of healthcare practitioners, such as endocrinologists, internists, and nurse practitioners, providing a holistic perspective on the topic. It is noteworthy that the study is not free of limitations. One of the limitations of the study is the small sample size, as only 30 healthcare professionals participated. This restricted sample size may limit the generalizability of the findings to a wider population of healthcare practitioners. Additionally, the study was conducted at a single healthcare institution, which may affect the representativeness of the results. The use of convenience sampling may introduce selection bias, as participants were selected based on their availability and accessibility. Furthermore, the study relied on self-reported data, which might be subject to recall and response bias. These limitations should be considered when interpreting the findings and applying them to other healthcare settings.

## Conclusions

In conclusion, this study revealed some knowledge gaps among healthcare professionals at KAMC in Jeddah regarding the preparation of patients with thyroid cancer for radioactive iodine therapy. Many participants struggled to answer knowledge-related questions, indicating a need for educational and training initiatives in this area. While some key aspects, such as rhTSH intake, low-iodine diet, and screening female patients for pregnancy, were correctly identified by many participants, yet critical aspects like excluding female patients from pregnancy screening and the duration for which male patients should avoid fathering a child were largely overlooked. These findings emphasize the importance of standardized protocols and guidelines to guide healthcare professionals in preparing patients for radioactive iodine therapy. By addressing these knowledge gaps and implementing targeted educational interventions, healthcare professionals can enhance patient safety, optimize treatment outcomes, and improve the overall effectiveness of radioactive iodine therapy in patients with thyroid cancer.
